# Proceedings of Fifth Annual Meeting of the Southern Dental Association

**Published:** 1873-09

**Authors:** 


### AMERICAN JOURNAL

OF

### DENTAL SCIENCE.

Vol. VII. THIRD SERIES--SEPTEMBER, 1873. No. 5.

### ARTICLE I.

### *Southern Dental Association.*

The Southern Dental Association, composed of members of the profession from all the Southern and some of the Northern States, commenced its fifth annual session, July 29th, at Raine's Hall, Baltimore. The following were the officers of the Association: President, Dr. H. M. Grant, Abingdon, Va.; 1st Vice-President, Dr. F. J. S. Gorgas, Baltimore, Md.; 2nd Vice-President, Dr. T. T. Moore, Columbia, S. C.; 3rd Vice-President, Dr. A. C. Ford, Atlanta, Ga.; Corresponding Secretary, Dr. O. J. Bond, Warren, S. C.; Recording Secretary, Dr. James F. Thompson, Fredericksburg, Va.; Treasurer, Dr. H. A. Lowrance, Athens, Ga.; Executive Committee, Drs. W. W. H. Thackston, F. J. S. Gorgas, John Mahoney, A. G. Bouton and A. C. Ford.

The convention was called to order about 10 o'clock, by the President, Dr. Grant, and the session was opened with prayer by Rev. Dr. Spangler. The roll of members was called by the Secretary, Dr. Thompson, and the following were found to be present: B. B. Alford, La Grange, Ga.; A. G. Bouton, Baltimore, Md.; F. Y. Clark, Savannah,

Ga.; Thomas F. Chupein, Charlestown, S. C.; G. G. Davidson, Lexington, Va.; E. Floyd, Fayetteville, N. C.; F. J. S. Gorgas, Baltimore, Md.; Hugh M. Grant, Abingdon, Va.; James Johnson, Staunton, Va.; J. H. Moore, Richmond, Va.; W. A. Miles, Norfolk, Va.; James F. Thompson, Fredericksburg, Va.; J. R. Walker, New Orleans, La.; Wm. C. Wardlaw, Abbeville, Va.; A. F. McLaine, New Orleans, La.

The President, Dr. Grant, then delivered the annual address. He referred to the favorable auspices under which the Southern Dental Association holds its present meeting. Only five years had elapsed since its organization, and one need only to look over the record of its transactions to be impressed with the vitality which it exhibits. Its discussions and reports evince a degree of scientific progress that is wonderful, and show that the impression is advancing out of the contracted sphere of mere artistic mechanics into the broader and higher field of thorough scientific investigation. The rapid strides which had been made by the dental profession at large during the past few years were then spoken of. There are now published seven dental periodicals, and eight dental colleges are now in prosperous existence. The establishment of a chair of Oral Surgery in the University of Pennsylvania, and the introduction of dentistry into the Medical department of Harvard College, the speaker claimed, might be taken as an indication of the spirit of the times. Dentistry should be classed with oral and opthalmic surgery, both of which were never considered apart from general medicine.

Dr. J. H. Moore was then appointed to act as Treasurer, that officer not being present.

Dr. James Johnston, Chairman of the Committee on Membership, reported the following applicants: Dr. James H. Harris, Dr. S. H. Williams, Dr. B. Coy, Dr. M. W. Foster, Dr. H. H. Keech, Dr. E. P. Keech, Dr. S. M. Field, Dr. A. McCloud, Dr. R. B. Winder, Dr. George Massamore and Dr. A. P. Gore, of Baltimore; Dr. R. F. Hunt and Dr. H.

B. Noble, of Washington; Dr. G. A. Sprinkle, of Madison Courthouse, Va.; Dr. J. R. Walker, from the Dental Association of New Orleans, La.; Dr. E. A. Bogue, N. Y.; Dr. G. H. Chewning, Fredericksburg, Va.; Dr. C. Little, Maryland; Dr. V. E. Turner, Raleigh, N. C.; Dr. E. L. Hunter, Enfield, N. C.; Dr. H. Henkle, Staunton, Va. All the applicants were elected.

Professor Gorgas submitted the report of the Executive Committee. He stated that Raine's Hall had been procured by the Faculty of the Baltimore College of Dental Surgery, as being better fitted for the purposes of the Convention than a lecture room in a college building. No arrangements could be effected with the railroad companies in reducing the rates of fare of the delegates to the Association. Professor Gorgas also stated that the proprietor of the Carrollton Hotel had promised to entertain the delegates at the rate of three dollars per day. From 8 to 10 A. M. daily, was set apart for clinical operations. The business hours were fixed from 10 o'clock to 1 o'clock in the morning, and from 3 to 6 o'clock in the evening.

Drs. J. R. Walker and H. B. Noble presented their credentials as delegates, and were admitted to seats in the Association. Certificates accrediting as delegates Drs. Samuel S. Williams and H. H. Keech, of Baltimore, were also read.

### Afternoon Session.

An afternoon session was held, when the credentials of Dr. S. H. Guilford, delegate from the Odontographic Society of Pennsylvania; Prof. James H. Harris, from the Baltimore College of Dental Surgery, and Dr. A. F. Herr, from the Pennsylvania State Dental Society, were presented and received.

During the afternoon session, Prof. Gorgas, from the Publishing Committee, submitted a report, showing the receipts and expenditures of the committee. The report was accepted and the expenses incurred were directed to be paid.

On motion of Dr. Thompson, the physicians and dentists of the city were invited to seats in the hall.

Dr. S. P. Cutler, of the Committee on Physiology and Surgery, sent a report, which was read by Prof. Gorgas. The report was an elaborate paper upon subjects of physiology and surgery. The paper elicited much interest, and Dr. Chupein moved a vote of thanks be tendered Dr. Cutler for the very able paper. Dr. Clark opposed such a vote being given, claiming that the paper was incomprehensible and impracticable, and did not cover the ground for which the committee was appointed. His objection to the vote of thanks was upon the ground that by giving such a vote the Association would be adopting the paper. The motion of Dr. Chupein was rejected.

Dr. Walker offered the following resolutions:

*Resolved by the Southern Dental Association in convention at Baltimore, Md.*, That the recent action of the railroad combination, in as far as it applies to the Dental, Medical, or Scientific Associations, is not only uncalled for but highly reprehensible.

*Resolved*, That gentlemen who attend as delegates upon such conventions do so at a great personal sacrifice for the benefit of humanity and the general public good, and that this influence tends greatly to advance the interests as well as the area of civilization, and by so doing to confer a positive benefit upon railroads, which depend upon the advancement and existence of civilization for their business and welfare.

*Resolved*, That railroad companies could well afford to pass such delegates to and from the places of meeting, and that their refusal to grant, at least upon the return route, passes, is placing a severe tax upon men who spend much time and labor for the public good, and which they are illy able to bare, and that such action by the railroads is as short sighted as it is arrogant and selfish.

Dr. Clark, of Savannah, Ga., delivered an address on Histology and Microscopy He said he differed from what was laid down in the books as to the cause of dental caries. It had been said that acids were the cause of it, but he contended, after a careful research, that caries were caused by parasites. Carbolic acid was a powerful agent for the destruction of these parasites, and it was because tobacco was a powerful disinfectant, and had the effect of destroying these parasites that it was a partial preserver of the teeth.

Prof. McLaine contended that Dr. Clark had taken the effect for the cause. He could not see how a parasite could enter the hard enamel of a tooth.

Drs. Foster and Walker gave their opinions on Dr. Clark's suggestion.

Prof. McQuillan was called upon, and said that he had just arrived from Philadelphia, and was not in a condition to speak upon the subject. He would remark that it is an undecided question whether parasites found on teeth belong to the animal or vegetable kingdom. Whether they are active agents in causing the decay of teeth he did not know. He has often examined them with microscopes and has seen them moving about.

The subject was then laid over for further discussion until to-morrow.

Dr. Chupein suggested that another hall be procured for the next meeting, as there was great difficulty in hearing the speakers in this hall, owing to the busy street on which it is located. The matter was then referred to the Executive Committee, who procured a smaller hall on the story below.

Drs. Williams, Walker and Harris, were added to the Executive Committee, to fill vacancies. Drs. Coy, Harris, James H. Johnston and Prof. McLain, were appointed to operate at clinics for Wednesday morning. Adjourned until Wednesday, 10 o'clock A. M.

### WEDNESDAY.—Morning Session.

The session was opened with prayer by Rev. Dr. Spangler.

Dr. James Johnson, chairman of the Committee on Membership, reported the following applicants, who were elected: Dr. John G. Wayt, of Richmond Dr. A. D. Cobey, Maryland; Dr. T. S. Waters, Baltimore; Dr. Bernhard Myer, Baltimore; Dr. W. G. A. Bonwill, of Philadelphia.

Dr. Clark stated that there were some delegates present from Pennsylvania and elsewhere, who had come to the meeting under a misapprehension as to the number of delegates that would be admitted from State societies, and he therefore moved that such delegates be admitted as members for this year, and for a longer period if they so desire. The motion was adopted.

Certificates of election as delegates from the Pennsylvania State Dental Society were presented by Drs. F. Hickman, J. Z. Hoffer, F. Welcher, S. Welchens, M. H. Webb and W. N. Amer; by John G. Wayt, from the Virginia State Dental Association, and by Prof. C. A. Kingsbury, from the Odontographic Society of Pennsylvania.

Profs. H. R. Noel and E. Lloyd Howard, of the Baltimore College of Dental Surgery, and Dr. McLain Tiffany, of the University of Maryland Medical School, were elected honorary members.

Dr. Walker moved that Dr. R. N. Laurence be dropped from the list of members, he having been expelled by the New Orleans Society.

Dr. Wardelow, of South Carolina, moved that a committee of three be appointed to investigate the justice of the charges preferred against him. Adopted.

The chair appointed Dr. Walker, of New Orleans; Dr. Wardlaw, of South Carolina, and Dr. Floyd of North Carolina, upon the committee.

On motion of Dr. Clark, the dental and medical students of Baltimore, were invited to seats on the floor.

Dr. Floyd moved that the Treasurer make up the list, and ascertain the names of those who have paid their dues and are entitled to seats on the floor, and to participate in the discussion.

Dr. R. B. Winder and Dr. S. H. Williams, of Baltimore, and Dr. R. B. Alford, of Ga., were appointed upon the committee on dental appliances and improvements.

Dr. Wm. H. Atkinson, of New York, read a very elaborate paper upon histology and microscopy. The paper was deeply scientific, and seemed to have been prepared with considerable care. Before proceeding the Doctor said he intended to say what he meant, and meant what he said. His sole object is to get at the truth, and if anything in his paper was ambiguous he begged to be interrupted, and requested to explain. Their only aim ought to be to get at the truth, though it take hold of the throne of the Infinite. His deepest conviction was that all are of one parentage, and have a right to claim one fraternity. He cared not whether men bless or curse, so they are in real earnest and desire to know the truth. Histology is most crippled, he continued, in the hands of its friends, who grasp at questions beyond their reach. The microscope has so enlarged the domain of knowledge that we prefer to investigate for ourselves rather than read the accounts of others.

After occupying about one hour in reading his production, he said he had just blocked out the way, set the stakes and driven the mule team through. The speaker caused some merriment at times by his style of delivery and seemingly quaint assertions. He was, however, closely listened to throughout, and much importance seemed to be attached to his views by the members of the association.

Dr. Atkinson concluded by saying that he was proud of his profession, as it has been reserved for it to discover the science of being. Advance objections, either in love or hate, so you get out of the conservation that has been handed down by false teaching.

Prof. McQuillan then spoke on the subject of oral parasites. He quoted from Dr. Watson to show that the parasites move in the skin, producing disturbances: each of them has its special domicile. In the mouth of man and lovely woman these parasites move and have their being. Whether they belong to the animal or vegetable kingdom is still an undecided question. When we get to the close boundaries of the animal kingdom we must remain in doubt to which the subject rightly belongs. Whether they are active agents in the decay and destruction of the teeth is also undecided. There is an analogy between the green substance found on damp stone and that which exerts an influence on the enamel. The monads are the smallest beings discernible under the microscope, but it is doubtful whether they are the smallest beings in existence. There is going on a constant destruction of beings by others struggling into existence in the animal, as well as the vegetable kingdom. The denticula has been observed on the decay of teeth.

It has been assumed that tartar has its origin in the parasites. An analogy has been attempted between the tartar on the teeth and the coral reefs which are considered as the work of parasites. He read a quotation from Herbert Spencer on this subject, and also referred to the services rendered to the profession by Dr. John Richardson, who established an apparent identity between the white and solitary corpuscles. The first element of decay is chemical decomposition.

Prof. Noel, of Baltimore, had never studied particularly the connection parasites had with dental caries; he had regarded caries as being caused by chemical decomposition, and when the enamel decayed he looked upon it as being caused by some inorganic element.

The Association then adjourned until 3 o'clock in the afternoon.

### Afternoon Session.

At the afternoon session the President announced that the subject of histology would be continued.

No one seeming willing to enter the contest, Dr. Atkinson arose and said he was pained to see so little interest taken in the subject, and that so many were fighting shy of it, thus leaving it to a few whose lives and reputation were set upon the discharge of the duty required of them. Dr. Atkinson then commenced another scientific discussion of histology.

Before going far he was called to order by Dr. Clark, who said, as he understood it, the subject of parisitie decay was the subject continued to the afternoon session. The chair ruled Dr. Atkinson in order, and time was granted him to proceed.

Dr. Atkinson said he liked what Dr. McQuillan had said upon the subject, but the latter did not put fire enough in his remarks. If Dr. McQuillan had put into his remarks the fire which nature had endowed him (the speaker) with, then the latter would shout hallelujah all the time. (Laughter.)

Dr. Atkinson continued until he was called to order by the chair on account of his having exhausted the allotted time. The discussion ended, and the paper read at the morning session by Dr. Atkinson, was received as the report of the Committee on Histology and Microscopy.

Dr. James B. Hodgkins, of Alexandria, Va., and Dr. Wm. Farmer, of Farmville, Va., were admitted as members of the Association.

Dr. W. C. Wardlaw, of South Carolina, from the Committee on Dental Therapeutics, read a report upon the subject. Therapeutics, he said, was the application of medicine, or means to the cure of disease, but when dental was added to it, the meaning of therapeutics was limited. He argued that the dentist was deprived of the means of observation from the fact that people generally apply to him, only to have their teeth extracted or filled, when they should in cases of dental neuralgia, &c., instead of applying to the general practitioner, apply to the specialist. Often cases belonging to the D. D. S., are placed in the hands of the M. D. The former ought to be oftener called into consultation with the latter.

Dr. Gorgas, of the Executive Committee, reported that they had examined the accounts of the treasurer, Dr. H. A. Lowrance, and found them correct. The account shows that there is remaining in the hands of the treasurer $346.10. Several bills, not included in the Treasurer's report, were reported by Dr. Gorgas and ordered to be paid. A dispatch was received by the Secretary and read, from Dr. Thomas Moore, of Columbia, S. C., 2nd Vice-President, who was prevented from attending the meeting on account of ill health, sending congratulations.

Dr. F. Y. Clark, from the committee on Operative Den tistry, read a report upon this subject. In treating the subject of the care of children's teeth, he urged the importance of children being thoroughly instructed in the use of the brush. There was not one in five, he said, who knew how to use the brush. It should be used three or four minutes upon the teeth, in order to insure thorough cleansing. He referred to the diversity of opinions among practitioners as to the kind of gold that should be used in filling, and the kind of instrument with which to operate. His advice to practitioners was to practice that with which they were familiar, until they find out for themselves something better.

Prof. McQuillan made some remarks in favor of contour filling. The Creator has made the teeth in the best form, and he desired to raise his voice against condemning contour filling, and he could not let the occasion pass without raising his voice in advocacy of contour filling. In some cases be believed that the treatment in cutting away the consumable part of the teeth, was not proper. When the patient is such that he will take care of it after it is filled, the operation is very desirable. He saw specimens of contour filling that have stood the test of years, and are in themselves works of art. He had some experience in it himself, and had successes as well as failures. He was in favor of Dr. Arthur's plan, but he must raise his voice against the condemnation by that author of contour filling.

Dr. Clark said that the contour filling is a very difficult operation, while in the other plan the machine invented by Dr. Morrison made the task comparatively easy.

Dr. Clark said his experience in contour filling was that it was a failure.

Dr. Walker said that the Association had met in Baltimore, where the first dental college in the world was established, expecting to receive a perfect flood of light, but he was astonished to find that the members of the Association from Baltimore who were capable of giving light kept their mouths closed.

Dr. Arthur being present, he was called upon to make a few remarks, but declined.

Dr. Walker was surprised that the many distinguished Baltimore dentists present who have given so much time and labor to subjects which absorb the dentist's mind, do not say anything in enlightening the members of the Association.

Dr. Winder thought that there was no special treatment which can be adopted by all operators alike.

Dr. Arthur then said that his plan was misunderstood by many on this floor. There are three methods of treating teeth. The first is that of combatting this prevaleni disease; the second the filling of the teeth, and the third in arresting the decay of the teeth. This last is older than any other method. In cases where the caries of the upper surface is inevitable, it can be arrested by a renewal of the teeth, and then it becomes advisable to separate them. No one will dispute that when two teeth are not attacked by the caries it is extremely difficult to determine when to remove the caries. What excuse can there be to induce men to wait until the caries is visible? for then the teeth are already destroyed. He would not dispute the results obtained in contour filling. If it is true that caries occur in consequence of close contact with teeth, then the operation is effective. If it is effective, how many are able to do it, and how many want to pay for it? We must not sneer at any operation; we must consider any method of treatment. The best men in the profession have adopted his (the speaker's) method; that is a gratifying fact, because it contributes to the welfare of the community.

Dr. Atkinson said that it was the misfortune of dentists that they hold on to their old opinions and refuse to accept what is new. Those among them that are regarded as novices are ahead of those that have toiled their life-long and maintained some dogma. He could not honor a man who stubbornly holds to his opinion, although new discoveries have been made in the science. Those people have no belief, only an opinion. Let us not go one against another, but let our sole aim be to discover the truth. He would like that in these conventions each one would work with zeal, so as to level every man up in his knowledge of the profession. According to the scriptural mandate we should "freely give and freely receive" of the rich treasures of our intellect. The hour of adjourning having arrived the Convention adjourned until Thursday morning at 10 o'clock.

Drs. J. H. Moore, F. J. S. Gorgas, and H. H. Keech, were appointed operators at the clinic for Thursday morning.

### THURSDAY—Morning Session.

The Association was called to order at 10 A. M. Rev. Dr. Spangler opening with prayer.

The discussion of the subject of operative dentistry, which was laid over from Wednesday, was resumed. Dr. McQuillan, of Philadelphia, said he had been charged with making an attack upon the paper submitted on Wednesday by Dr. Clark, chairman of the committee on operative dentistry. He disclaimed any such intention—He had a head of his own, and claimed the right to express his views. It had been said during the discussion of this subject that certain things did not pay. Gentlemen, the speaker said, it always pays to do the right thing. [Applause.]

Dr. Clark said this doing the right thing is what every honest man ought to do, and is what every man within the sound of his voice is trying to do. But what is the right thing? That is the rub. Honest men have used mercurial filling, thereby ruining the teeth and entailing misery upon humanity; they believed they were doing the right thing. Many believe contour filling the right thing; the speaker honestly thought it was not; he was now going down the other side of the hill, and he thought that it was the wrong thing. Contour filling he did not believe would save the teeth. It was the duty of the dentist to save the teeth; do what they will—fill them with putty, if that would accomplish the object—but at all events it was the duty of the dentist to save the teeth. [Applause.] He did not care to see a gold sign show itself every time the lips were parted. It costs from $100 to $200 for such a filling, and men receiving a salary of $1,000 per year were required to pay such a price, when the tooth could be saved at little expense. Is that the right thing? He contended that the cheapest and best way is the right thing.

Dr. Walker maintained that contour filling will save the teeth; it might fail sometimes. The duty of future dentists, the speaker said, would be to give advice, when they would be employed by the year by families, instead of tinkering as they do now.

Dr. Foster said that the thorough dentist looked to the welfare of the whole mouth. Parents intrusted their child to the dentist, with the instruction to do what the dentist deemed best, not only fill the teeth, but place the mouth in the best condition.

Dr. S. Welchens, of Pa. then read a paper entitled "Darwinism *vs.* Six Year Molars." He strongly refuted the doctrine of Darwin that man had its origin in a fish, which had progressed to a frog, to a monkey, to a man, &c. It was as impossible, he said, for a frog to be changed into a monkey as it was to change a mosquito into an elephant or a dog into a horse.

The removal of the six years' molar, the speaker said, has an injurious effect.

Prof. Stellwagen said that the dentists must be governed by principle. If they are not guided by it they may just as well send for a number of coolies, and they can easily be taught the handicraft. A self cleansing surface must be kept, but this cannot be done by simply separating the teeth. Whenever it is possible, the wedging of the food between the teeth ought to be prevented. When the food will wedge between the teeth it will cause decomposition. When the decay is extensive he could not understand how any one could object to contour filling. There are cases when the extraction of the six years' molar before it made its appearance on the gum is advisable. With regard to wedging, he would always cut away before attempting to drive away.

Prof McQuillan again said that he was in favor of eclectic practice. He was not here to dictate terms or take advantage of his position.

Dr. Chupein thought that whenever self-cleansing can be accomplished it is well to be done.

Dr. Guilford said that the highest aim of the dentist should be the preservation of the teeth. He believed in the judicious extraction of the six year molar. You must keep the teeth from getting crowded. In regard to the judicious extraction of the six year molar, and letting the second molar come into its place, he often succeeded. When a tendency is observed among children to irregularities of teeth, it is best to anticipate by giving the teeth plenty of room. In nine cases out of ten when the arch is expanded a deformity is produced. In cases when the upper jaw shows a tendency to protrude, he would extract the teeth in the lower jaw, and *vice versa.*

Dr. Guilford then addressed himself to the subject of the Degeneration of the Human Race, and asked the question, has the Human Race degenerated? The speaker answered his question in the affirmative, and said that the human frame now is not the same as it was one thousand years ago. A friend of the speaker had examined skulls 4,000 and 400 years old, and there was a great difference between the two, unfavorable to the latter.

Dr. Chupein differed with Dr. Guilford on this subject. The reason that the skeleton of the Egyptian was more perfect in its form was that the Egyptians were not a migratory race. In this country there are large numbers of people of various nationalities, and owing to the intermarriage between persons of different races you can account for the dissimilarity of the jaws and teeth. A German has wide teeth and wide jaws; an Italian has narrow jaws. Should persons of these two races marry together the child of this union may partake of the jaws of the mother and teeth of the father.

Dr. Walker did not believe in the deterioration of the human race. We have no reliable statistics of the duration of life of human beings in the olden times, but in modern times we have, and when we compare the statistics of the present century with former years, we will find that the duration of life is rather on the increase. The physical development is controlled entirely by local circumstances. There is no race superior or equal to that now in America.

Dr. Arthur said that there are cases when the extraction of teeth becomes a necessity. In cases where it is advisable to give room, it is better to extract the bicuspid than the molar teeth.

Dr. Clark advocated the expansion of the jaw, and was opposed to the indiscriminate extraction of bicuspid teeth, for if that system was continued there would eventually be none to remove, just as if you cut off the tails of dogs for a few years, there would eventually be no tails to cut off. It would also result in disfigured faces, &c.

The subject was further discussed by Drs. Hickman, Webb, Guilford, Walker and Noble, and the paper submitted on Wednesday by Dr. Clark was received as the report of the committee on Operative Surgery.

A letter was read from Dr. W. H. Eames, President of the Mississippi Valley Dental Association, Dr. G. A. Bowman, President of the St. Louis Dental Society, Dr. Price, President of the Missouri Dental Association, and Dr. Wm. N. Morrison, inviting the Association to hold their next meeting in St. Louis, on the first Tuesday in March, 1874, where the Mississippi Valley Dental Association and the Missouri State Association are to hold joint meetings. The matter was laid over. A letter was also read from Dr. W.

W. H. Thackston, of Va., regretting his inability to be present on account of indisposition and sickness in his family.

Prof. T. O. Summers of the Southern University of Greensboro', Albama, and Prof. Joseph Jones, of New Orleans, were elected honorary members of the Association. The chair stated that he desired the members to designate who were going to the session of the American Dental Association to be held at Put-in-Bay, so that he could appoint the delegates from this Association.

The subject of mechanical dentistry was taken up, but no member of the committee upon the subject being present, no report upon the same was made. Some discussion, however arose upon a resolution offered by Dr. James Johnston, "that this Association discountenance the use of the rubber for a base for dental purposes, at least until after a decision of the suit now pending in the Supreme Court."

Dr. Walker thought it would be to the interest of the members of the Association to abandon the use of the rubber.

Prof. McLain believed the use of the rubber lowered the standard of the profession mechanically, but the Association had no right to abridge the privileges of any of its members.

A motion was made to lay the resolution on the table, and the motion was carried.

The Association adjourned until Friday morning, the afternoon being occupied in an excursion.

Before adjourning for the day the Association was invited to enjoy an excursion down the river and a banquet at Pavilion Retreat. The members of the Association, escorted by resident members, marched in procession to Light St. wharf, headed by Minnick's Band, where they embarked on the steamboat Cyrus P. Smith, and were soon steaming down the river. The steamer arrived at Pavilion Retreat about 4 o'clock, and after spending a short time upon the beautiful grounds, repaired to the eating saloon out in the grove, where they partook of a soft crab and chicken dinner, served in good syle by the proprietor, Mr. Harry McGowan. The lateness of the hour and the exhilarating air of the river seemed to have sharpened the appetites of the excursionists, who attacked the viands with remarkable relish. There was a commendable absence of anything spirituous at the dinner, though there was a profusion of fluids on board the boat. The absence of such exciting influences seemed to have had the effect of doing away with the long array of toasts and correspondingly long responses usually inflicted upon such occasions.

The party re-embarked on board the steamer about 6 o'clock, and after returning to the Patapsco river, proceeded some distance down the Chesapeake bay, and arrived in the city shortly after 10 o'clock. The excursionists after landing again formed into line, and accompanied by the band, proceeded to the Carrollton Hotel, where many of the members of the Association were stopping.

### FRIDAY—Morning Session.

The President called the Association to order at 9 A. M. followed by prayer.

On motion all members in arrears for two years were dropped from the roll

The discussion on mechanical dentistry was resumed, and the subject of plastic materials considered. It was generally admitted that the celluloid base was a failure, although the pyroxyline had produced better results.

The construction of artificial palates was then discussed, and Dr. Grant said as to the process of manufacture he would refer for description to the last edition of Harris' Dental Surgery, which is minute in detail. He thought the semi-vulcanized rubber should be worked in plaster, or a soft delicate texture used. Models are made of type metal and the materials vulcanized in a temperatureof 310 degrees

Dr. Johnston thought there was a want of uniformity in the material composing the base which caused secretions, and advised the use of celluloid rather than vulcanized rubber. Upon this question there was considerable discussion-the majority of opinion being against the use of celluloid.

The subject of irregularity of the teeth was then resumed, and Dr. G. H. Chewning, of Va., presented a plaster model of a case of irregularity of the superior central incisors, which he referred to Prof. Gorgas, who described the condition of the mouth, and the method for the correction of the abnormally placed teeth.

Dr. G. F. S. Wright, of S. C., chairman, submitted the report of the Committee on Dental Literature, which contained some very good suggestions, on which, however, no action was taken by the Association. Dr. Wright referred to the new publications of the past year, such as Tome's Dental Surgery, 2nd edition, and Cole's Treatise on Dental Mechanics, both English works. The subject of dental education caused a protracted and interesting debate.

Dr. Arthur said that this subject is one of great interest to every member of the profession. Every individual who comes to their Associations expects to be benefited by his attendance. It is too common for individuals to look to their own benefit without consideration of others. While we take care of ourselves we must also see to the good of others. If we wish to escape the evils which threaten our profession, we must scrutinize the manner in which our colleges are conducted. If any of these institutions turn out a man who is incapable to perform the services of the profession, let the professors of those colleges be held responsible. It is in the power of the dental profession to remedy this evil, and to establish a higher standard by cautioning students against colleges which are improperly conducted. They can establish an institution where the standard of education can be high, and where the professors may be held accountable

Prof. Noel remarked that he had attended the sessions of the American Medical Association, where this subject was continually discussed, referred to committees and then postponed. There is but one institution in the country where the standard was maintained before the late war, and that was the University of Virginia. It is almost impossible to maintain the standard in our schools and colleges. The fault lies in the American people, and the rapidity with which they push everything through. No education will be of any consequence which is not slowly and steadily acquired. Another defect in the colleges is the fixing of the remuneration of the professors of the colleges, according to the number of pupils in the institutions. He thought that the Austrian method, giving to each professor a certain salary, was the best plan.

Again, the men who study dentistry are not those who have travelled extensively and have had a mental training. The colleges ought to supply the deficiency and teach them not only how to use their hands, but also their mind. The study of anatomy, chemistry, and physiology ought to be adopted. There is no success which is not obtained by hard, earnest labor.

Another evil in the system of colleges is lecturing against time. He always prepared his lectures before entering the lecture room, and each year he had reason to be proud of his school.

Prof. McLain, of New Orleans, said that two years is an insufficient time for any student to complete his studies. It would be a desirable plan to lengthen the term to three or four years.

Dr. Clark said that he had no partiality or prejudice toward any college, but it must be said that the dental education is disreputable to the profession. Why not seek some better road when we know that the present system is imperfect. It is not the fault of the profession, but rather the dental colleges. The Association has sufficient influence and enough money to endow a university, as the professors of existing institutions are not willing to resign their positions for the good of the profession. The time has come when the Association will have to endow such an institution, as dental education has not kept pace with other improvements.

Dr. Clark then called for the report of the committee appointed at the last meeting to enter into negotiations with the dental colleges so as to bring them under the control of the Southern Dental Association; and stated that one gentlemen he knew of was willing to pay two thousand dollars towards endowing a dental college.

Dr. Grant, chairman of the committee (Prof. Gorgas in the chair,) read the report, which stated that the committee had written to the Baltimore College of Dental Surgery and to the New Orleans Dental College. Both of them rejected the request of the committee. The Baltimore College offered to the Association the endowment of a professorship in histology and microscopy. Nothing has therefore been effected by the action of the committee.

Professor McQuillen, of Pennsylvania, did not think that there were too many colleges. There are at least one thousand members added every year to the profession. The point is, are the colleges properly managed? It may be true that the best plan is to endow these institutions, but where is the man in the profession to endow them. The necessities of the professors are such that they cannot devote their entire time to teaching. The speaker thought that it would redound to the advantage of the profession if a State law was passed compelling each individual desiring to enter the profession to study a number of years.

Professor McLain defended the step taken by the New Orleans College, and stated that if any offense was taken from it, he was very sorry, as such was not intended.

Professor Gorgas, Dean of the Baltimore College, said that the proposition of the Association had been carefully considered and respectfully answered by the Faculty of the Baltimore College of Dental Surgery. He asked if the Association was able to assume the burden of supporting a College? Are the members willing to be subjected to a heavy tax in its behalf? Is any gentlemen present, able, and if able, willing to be subjected to a heavy annual tax to support any institution? The Southern Dental Association at the commencement of the present session, consisted of one hundred and twenty-seven members; and at least one hundred thousand dollars would be required to properly endow a dental college, in addition to all that could be received from students. As one of the founders of the Southern Dental Association, one of the committee who framed its constitution and by-laws, and its first Recording Secretary, he took great interest in its success, and would use his best efforts to have it succeed; therefore, any proposition coming from this Association through him, would be zealously advocated.

The Faculty of the Baltimore College of Dental Surgery placed it in the power of the Association to establish one professorship, and then by degrees to have control over the college by creating new professorships, or endowing old ones, as their means to do so from time to time justified. To place the Baltimore College in the hands of the Association at this time, would result in a failure to support it, and cause its downfall. He had no aspiration for office in the Association, was not a candidate for any office, and hence was honest in his endeavors to do justice to both the Association and the College he represented.

He also stated that in his opinion, the objections against the proposition made to the Association by the Baltimore College, arose from the filling of the chair, it was proposed to endow, by the Faculty, thereby destroying the aspirations of some members of this Association who desire to become professors in a college, when they do not possess the qualifications necessary for such a position. Again, almost every one who complains of the colleges, is ignorant of their course of study and management, judging them by a few isolated cases occurring years ago when the country was convulsed by civil war, and the majority of the members of the Faculties absent in the army, and when a suspension would have forfeited their charter, and entailed the downfall of the institution by the persecution of the party then in power.

Dr. Arthur said that the action of the Association in appointing the committee was a mistake. Throw away the sentimental regard for your alma mater, and look alone to the elevation of the profession.

Dr. Walker thought that what was wanted is the combination of all dental associations in America in establishing a dental college.

Prof. Stellwagen spoke in opposition to the endowment of any College. He claimed that the excellency of the universities of Germany consists in the devotion and self-sacrifice of the faculties, the members of which, although receiving a small income, are independent of any masters. Endowments will not make a good college. Every one must individually do all he can to advance his profession. The moment the schools are endowed, the faculties must look to the manufacturer and the dental author, grown wealthy by his wares and books, and you will bring about a servitude which any honest man would shrink from as contemptible.

Dr. Coy then moved to re-commit the report to the committee, in order that a proposition from an institution in an embryo state might be received; which motion was ruled out of order by the President.

Dr. Johnston, of Virginia, moved that the letter read at the morning session, received from the New Orleans College of Dentistry, relative to consolidation with Baltimore, be not accepted.*

The motion was withdrawn after a few remarks by Dr. Grant, who moved that the whole matter be laid upon the table, which was carried.

Dr. Robert Arthur, was elected an active member, and Dr. C. F. Grindall, Dr. J. Wm. Welsh, of Baltimore, and Dr. John H. Crawford, of N. C. were elected members of the Association.

The meeting then adjourned until 3 P. M.

---

* We publish in the present number, the answers of the Baltimore College of Dental Surgery, and the New Orleans Dental College, to the Southern Dental Association.

### Afternoon Session.

The Association reassembled in Raine's Hall at 3 o'clock in the afternoon, there being but a slim attendance; so many of the delegates having left for their homes, and all the important business being over, that scarcely a dozen attended the afternoon session, which completed the work of the Association for this annual meeting.

Dr. Winder, of Baltimore, made a few remarks upon the Baltimore College of Dental Surgery; but as no member of the Faculty of this institution was present to reply to him, his remarks elicited no response, and the further discussion of the subject was postponed.

Dr. James F. Thompson, Recording Secretary, read a paper from Dr. S. J. Cobb, chairman of the Committee on Voluntary Essays, treating upon colleges and operative dentistry.

The report of the Committee on Dental Appliances and Improvements, was read by Dr. Foster, of Baltimore, and adopted.

Prof. McLain read an able paper on the causes of decay of the human teeth, which was accepted.

St. Louis was selected for the next place of meeting of the Association, on the last Tuesday in July, 1874.

A resolution offered by Dr. Clark to change the name of the Southern Dental Association to the National Dental Association, was called up, debated, and lost.

The following resolution offered by Dr. Thompson, was adopted:

*Resolved*, That a vote of thanks from this Association is due to Dr. Samuel S. White, of Philadelphia, for his untiring and gratuitous efforts in behalf of the dental profession, in successfully resisting the illegal action of the Goodyear Dental Vulcanite Company in the Gardner case.

*Resolved*, That we hereby express our unqualified confidence in his integrity, and in his ability to conduct to a successful issue any litigation which he may undertake in our behalf.

An election of officers for the ensuing year resulted in the following gentlemen being declared elected, viz: President, Dr. Robert Arthur, Baltimore; 1st Vice-President, Dr. Theodore F. Chupein, Charleston, S. C.; 2nd Vice-President, Dr. James Johnston, Va.; 3rd Vice-President, Dr. E. Floyd, N. C.; Corresponding Secretary, Dr. Samuel M. Field, Md.; Recording Secretary, Dr. James F. Thompson, Fredericksburg, Va.; Treasurer, Dr. J. Hall Moore, Richmond, Va.; Executive Committee, Dr. J. R. Walker, New Orleans; Dr. W. N. Morrison, St. Louis; Dr. W. G. Redman, Louisville, Ky.; Dr. Jonathan Taft, Cincinnati O,; Dr. S. J. Cobb, Nashville, Tenn.

The President elect was escorted to the chair, who thanked the Association for the honor conferred upon him. The retiring President, Dr. Grant, also made a few happy remarks.

A unanimous vote of thanks was tendered to Dr. Grant for the efficient and impartial manner in which he had discharged his duties; also to Colonel Coleman, of the Carrollton Hotel, for reduction in charges.

Although the Faculty of the Baltimore College of Dental Surgery furnished Raine's Hall for the meetings of the Association, no notice was taken of this fact by the few members present at the closing session; votes of thanks were tendered to others, however, for smaller favors.

---

### ARTICLE II.

### *The Southern Dental Association and the Dental Colleges.*

Southern Dental Association, at the Richmond Meeting, 1872.

